# Engraftment Outcomes after HPC Co-Culture with Mesenchymal Stromal Cells and Osteoblasts

**DOI:** 10.3390/jcm2030115

**Published:** 2013-09-23

**Authors:** Matthew M. Cook, Michael R. Doran, Katarina Kollar, Valerie Barbier, Ingrid G. Winkler, Jean-Pierre Levesque, Gary Brooke, Kerry Atkinson

**Affiliations:** 1 Stem Cell and Regenerative Medicine Group, Biological Therapies Program, Mater Research Institute, University of Queensland, TRI Building, 37 Kent Street, Woolloongabba, Queensland 4102, Australia; E-Mails: michael.doran@qut.edu.au (M.R.D.); katekollar@gmail.com (K.K.); gpbrooke@gmail.com (G.B.); m.atkinson1@uq.edu.au (K.A.); 2 School of Medicine, University of Queensland, 288 Herston Road, Herston, Queensland 4006, Australia; E-Mail: jplevesque@mmri.mater.org.au; 3 Institute of Health and Biomedical Innovation, Queensland University of Technology, 60 Musk Avenue, Kelvin Grove Urban Village, Kelvin Grove, Queensland 4059, Australia; 4 Stem Cell and Cancer Group, Biological Therapies Program, Mater Medical Research Institute, Level 3, Aubigny Place, Raymond Terrace, South Brisbane, Queensland 4101, Australia; E-Mails: vbarbier@mmri.mater.org.au (V.B.); iwinkler@mmri.mater.org.au (I.G.W.)

**Keywords:** haematopoietic stem cells, mesenchymal stromal cells, osteoblasts, *ex vivo* expansion, haematopoietic reconstitution

## Abstract

Haematopoietic stem cell (HSC) transplantation is an established cell-based therapy for a number of haematological diseases. To enhance this therapy, there is considerable interest in expanding HSCs in artificial niches prior to transplantation. This study compared murine HSC expansion supported through co-culture on monolayers of either undifferentiated mesenchymal stromal cells (MSCs) or osteoblasts. Sorted Lineage^−^ Sca-1^+^ c-kit^+^ (LSK) haematopoietic stem/progenitor cells (HPC) demonstrated proliferative capacity on both stromal monolayers with the greatest expansion of LSK shown in cultures supported by osteoblast monolayers. After transplantation, both types of bulk-expanded cultures were capable of engrafting and repopulating lethally irradiated primary and secondary murine recipients. LSKs co-cultured on MSCs showed comparable, but not superior, reconstitution ability to that of freshly isolated LSKs. Surprisingly, however, osteoblast co-cultured LSKs showed significantly poorer haematopoietic reconstitution compared to LSKs co-cultured on MSCs, likely due to a delay in short-term reconstitution. We demonstrated that stromal monolayers can be used to maintain, but not expand, functional HSCs without a need for additional haematopoietic growth factors. We also demonstrated that despite apparently superior *in vitro* performance, co-injection of bulk cultures of osteoblasts and LSKs *in vivo* was detrimental to recipient survival and should be avoided in translation to clinical practice.

## 1. Introduction

Haematopoietic stem cell (HSC) transplantation is a curative treatment for a number of haematological malignancies, bone marrow aplasia, congenital haemoglobinopathies and immunodeficiencies [1,2]. Umbilical cord blood (UCB) transplantation is a promising alternative to bone marrow (BM) reconstitution for those who lack a human leucocyte antigen (HLA)-matched family member or a living unrelated donor [3]. Allogeneic UCB transplantation has been shown to elicit less frequent, and less severe, graft-*versus*-host disease (GVHD) than allogeneic marrow or mobilised peripheral blood stem cell transplantation [4,5,6,7,8,9,10]. However, the transplantation of UCB-derived HSCs is often associated with delayed engraftment, especially in adult recipients, mainly due to the small number of HSCs that can be recovered from a single cord. As HSC transplant outcomes are dose-dependent [11], it has been hypothesised that clinical outcomes could be significantly enhanced through *ex vivo* expansion of UCB-derived HSC prior to transplantation. 

Numerous studies describe on-going efforts to characterise the stromal support cell composition of the HSC BM niche [12,13,14,15,16,17,18,19,20]. There is mounting evidence that cells of the osteoblast lineage, namely osteoprogenitors or mesenchymal stromal cells (MSCs) likely play the most influential supportive roles [21,22,23] together with endothelial cells which have a critical role in HSC maintenance and proliferation in vascular HSC niches [24,25,26]. The first successful efforts to mimic this complex signal milieu, resulting only in transient *in vitro* HSC maintenance, were reported by Dexter and colleagues [27,28]. In these studies unselected populations of stromal and haematopoietic cells from whole BM were co-cultured. It is now well established that cell-cell contact between HSCs and BM niche stromal cells is essential for HSC regulation [29,30,31,32]. Therefore, like the studies by Dexter *et al.*, co-culture of HSCs in direct contact with a supportive stromal cell population remains a commonly used *in vitro* model system and expansion platform. 

Recent Dexter-type co-cultures have utilised osteoblast-lineage cells, as supportive feeder layers for *in vitro* HSC maintenance and/or expansion [15,19,33,34]. Nakamura and colleagues [34] successfully co-cultured LSK Flt-3^+^ HSCs with fresh MSCs thought to be pre-osteoblasts on the basis of Sca-1 and Alcam-1 expression. Likewise, Zhu and colleagues [35] co-cultured Lin^−^ Sca-1^+^ HSCs with osteoblasts differentiated as in this study, and released the HSCs with collagenase-trypsin treatment before transplantation. Similarly, a number of studies have shown the expansion of phenotypic HSCs when MSCs are used as feeder layers [36,37,38,39]. 

In an effort to elucidate the relative supportive capacity of undifferentiated MSCs *vs.* differentiated osteoblasts we used a murine system to directly compare the *in vitro* expansion potential of a purified population of HSCs on undifferentiated MSCs or on osteoblast feeder layers.

## 2. Experimental Section

### 2.1. Mice

C57BL/6 mice (purchased from the Australian Animal Resource Centre) or inbred C57BL/6 transgenic for green fluorescent protein (GFP) under the control of the ubiquitin promoter (C57BL/6-GFP) were used. All animal experiments were approved by the University of Queensland Animal Ethics Committee.

### 2.2. Isolation of LSK and MSC Populations

LSK and MSC populations were isolated from C57BL/6 or C57BL/6-GFP mice as previously described by our group [40]. All experiments involving MSCs were performed at passage 8–12. LSKs and MSCs were characterized by morphology, cell surface phenotype and functional capacity as previously published by our group [40]. 

### 2.3. Osteogenic Induction of Undifferentiated MSCs

MSCs were induced into the osteogenic lineage *in vitro* as follows: 2 × 10^4^ MSCs were seeded in 24-well plates, grown to confluence and cultured for 21 days in Dulbecco’s Modified Eagle Medium (DMEM) supplemented with dexamethasone (0.1 μM), β-glycerol phosphate (100 mM), l-ascorbate-2-phosphate (10 mM), calcium chloride (4 mM), 10% fetal calf serum (FCS) and gentamycin (40 μg/mL, Pfizer, New York, NY, USA). These were fixed in 4% paraformaldehyde (PFA) and stained for the presence of calcified osteoid deposits with Alizarin red S solution [40,41]. Undifferentiated MSCs and MSCs induced into osteoblasts were further characterised according to their gene expression of HSC niche markers including angiopoietin 1 and 2, stem cell factor, jagged-1 and stromal cell-derived factor 1 (CXCL12).

### 2.4. Flow Cytometry

Cell sorting and immunophenotype analysis was performed by flow cytometry using fluorochrome-labeled rat-anti mouse monoclonal antibodies (all at 1–2.5 μg/mL) as follows: c-kit allophycocyanin (APC; 2B8; BD, Franklin Lakes, NJ, USA), Sca-1 phycoerythrin cyanine-7 (PE Cy7; D7; BD), CD45 APC (30-F11; BD), CD31 PE (MEC13.3; BD), CD44 PE (IM7; BD), CD11b PE (M1/70; BD), F4/80 Pacific Blue (BM8; eBioscience, San Diego, CA, USA), Gr-1 APC Cy7 (RB6-8C5; BD), CD45R/B220 Pacific Blue (RA3-6B2; BD) and CD5 APC (53–7.3; BD). A biotinylated lineage cocktail (containing CD5 (53–7.3; BD), CD45R/B220 (RA3-6B2; BD), Gr-1 (RB6-8C5; BD) and F4/80 (BM8; eBioscience)) was also used for staining of haematopoietic cells with streptavidin Pacific Blue (Invitrogen, Carlsbad, CA, USA) secondary staining. Cell sorting was performed by fluorescence-activated cell sorting (FACS) on a FACS-ARIA (BD) and immunophenotyping was performed on an LSRII (BD) with results analysed in FlowJo Version 7.5 (Tree Star, Ashland, OR, USA).

### 2.5. RNA Extraction and PCR

RNA from was extracted with a Qiagen RNeasy Mini Kit (Qiagen, Venlo, Netherlands) using the manufacturer’s protocol. RNA was pre-incubated with DNase I (Invitrogen) and reverse transcription was performed with oligo-dT and Superscript III (Invitrogen) as described in the manufacturer’s instructions. Quantitative real-time polymerase chain reaction (qRT-PCR) was performed on cDNA using ABsolute™ QPCR SYBR^®^ Green (ABgene, Waltham, MA, USA). Product size and primer sequences used are shown in Table 1. 

**Table 1:** Primers used for characterisation of MSC and osteoblasts (Ob).

Primer	Sequence	Fragment size
GAPDH-(F)	5′-TGGTGAAGGTCGGTGTGAACG-3′	105
GAPDH-(R)	5′-CAATGAAGGGGTCGTTGATGGC-3′	
RUN2X-(F)	5′-CCACCTTTACCTACACCCCG-3′	89
RUN2X-(R)	5′-GGTGGCAGGTACGTGTGGTAGT-3′	
Osterix-(F)	5′-AGCTCACTATGGCTCCAGTCC-3′	21
Osterix-(R)	5′-GCGTATGGCTTCTTTGTGCCT-3′	
Osteocalcin-(F)	5′-TTCTGCTCACTCTGCTGACCCT-3′	22
Osteocalcin-(R)	5′-CCCTCCTGCTTGGACATGAA-3′	
Angiopoietin 1-(F)	5′-CAAATGCGCTCTCATGCTAA-3′	162
Angiopoietin 1-(R)	5′-ATGGTGGTGGAACGTAAGGA-3′	
Angiopoietin 2-(F)	5′-CCATCTTCTCGGTGTTGGAT-3′	194
Angiopoietin 2-(R)	5′-TCCAAGAGCTCGGTTGCTAT-3′	
Stem Cell Factor-(F)	5′-GCTACCCAATGCTGGGACTA-3′	207
Stem Cell Factor-(R)	5′-CCGCAGATCTCCTTGGTTT-3′	
Jagged-1-(F)	5′-AGTAGAAGGCTGTCACCAAGCAAC-3′	113
Jagged-1-(R)	5′-AGAAGTCAGAGTTCAGAGGCGTCC-3′	
SDF-1-(F)	5′-TGCCCTTCAGATTGTTGCACGG-3′	67
SDF-1-(R)	5′-ATTTCGGGTCAATGCACACTTGTC-3′	

### 2.6. Co-Culture Assay

MSCs were seeded into a 24-well plate and allowed to reach confluence. Osteoblasts were induced from MSC as described above and used for experiments after 2–3 weeks of differentiation. 1 × 10^3^ purified LSKs (500 cells/cm^2^) were seeded on top of stromal cells in static cultures for 7 days in BioWhittaker X-vivo 10 medium (Lonza, Basel, Switzerland) supplemented with 20% FCS and gentamycin (40 μg/mL). After this period the resultant cell populations were removed from culture, counted and analysed by flow cytometry for lineage-specific haematopoietic markers and LSK markers. 

### 2.7. Haematopoietic Reconstitution Assays

Female C57BL/6 recipient mice were administered a split dose of total body irradiation (2 × 550 cGy) 3 h apart via a self-contained Gammacell 40 Exactor (GC-40E) irradiator using a ^137^caesium radiation source. Twenty-four hours after irradiation, recipients were intravenously injected via the retro-orbital sinus with the appropriate cell populations resuspended in saline supplemented with 2% heat-inactivated FCS and DNase I (10 μg/mL, Roche, Basel, Switzerland). All mice were monitored by weight and clinical scoring over the first 28 days of the experiments and drinking water was supplemented with antibiotics and antifungal agents for the first 14 days. For analysis of functionality of the initial purified LSK population, BM cells and LSKs were isolated as above from C57BL/6-GFP mice. The c-kit depleted cells were sourced from the negative fraction of c-kit magnetic separation (cells devoid of HSCs) and the vehicle group received cell excipient only. For competitive repopulation transplants, 200 GFP^+^ LSKs or the equivalent number of cells produced in culture from 200 LSKs, were used as the donor population together with 4 × 10^5^ whole bone marrow (BM) cells from C57BL/6 as the competitor cells. Where performed, MSCs and osteoblasts were physically removed from harvested co-cultures prior to transplantation using flow sorting for GFP^+^ haematopoietic cells. For secondary transplants, BM was harvested from primary recipients, pooled and 2 × 10^6^ cells transplanted into lethally irradiated secondary recipients. All treatment groups were monitored for health and well-being by daily scoring. Tail bleeding was performed 4, 6, 8, 10 and 12 weeks post-transplant and flow cytometry analysis of GFP was used to detect the level of donor (GFP^+^) and competitive (GFP^−^) total cells, myeloid cells (CDllb^+^) and B cells (CD45R/B220^+^) in the recipient blood. T cell engraftment was not examined because of the known ability of recipient T cells to survive high-dose total body irradiation. Donor populations were deemed to be engrafted if greater than 1% GFP^+^ cells were observed in blood or BM. Blood, BM and spleen counts were performed on a Sysmex KX-21N Cell Counter (Sysmex Corporation, Kobe, Japan).

### 2.8. Genomic DNA Extraction and Detection of GFP by qRT-PCR

Snap frozen tissue was crushed into a powder using a pestle and mortar on dry ice and digested by overnight incubation with Proteinase K at 56 °C. The genomic DNA was then extracted using a QIAamp DNA Mini Kit (Qiagen) according to the manufacturer’s protocol (RNA was removed by RNase treatment). The genomic DNA was assessed for the presence of genomic GFP using the primers and fluorescent probe as follows: 5′end: CTGCTGCCCGACAACCA. 3′end: TGTGATCGCGCTTCTCGTT fluorescent probe 5′end: CCCAGTCCGCCCTGAGCAAAGAC. The number of GFP^+^ cells was then calculated using a standard curve of the amount of GFP genomic DNA from known cell numbers.

### 2.9. Statistical Analysis

Either a Mann-Whitney test or Kruskal-Wallis test with Dunn’s Multiple Comparison post-test was used to test statistical significance between groups. This was performed using GraphPad Prism version 4.03 for Windows (GraphPad Software, La Jolla, CA, USA). Results were deemed statistically different if *p* < 0.01. All continuous data were expressed as mean ± 1 standard deviation (SD).

## 3. Results and Discussion

### 3.1. Characterisation of Murine LSKs and MSCs

LSK and MSC populations were characterised as per Cook *et al.* (2012) [40]. Briefly, LSKs were shown to be enriched for a true repopulating population of HSC by transplantation of 200 LSKs into lethally myeloblated recipients and achieving 100% survival for >16 weeks (data not shown). MSCs had a classical fibroblast morphology and were Sca-1^+^, CD44^+^, CD45^−^, CD11b^−^ and CD31^−^. MSC were also shown to be able to differentiate into osteoblasts, adipocytes and chondrocytes [40,41] (data not shown).

**Figure 1:**
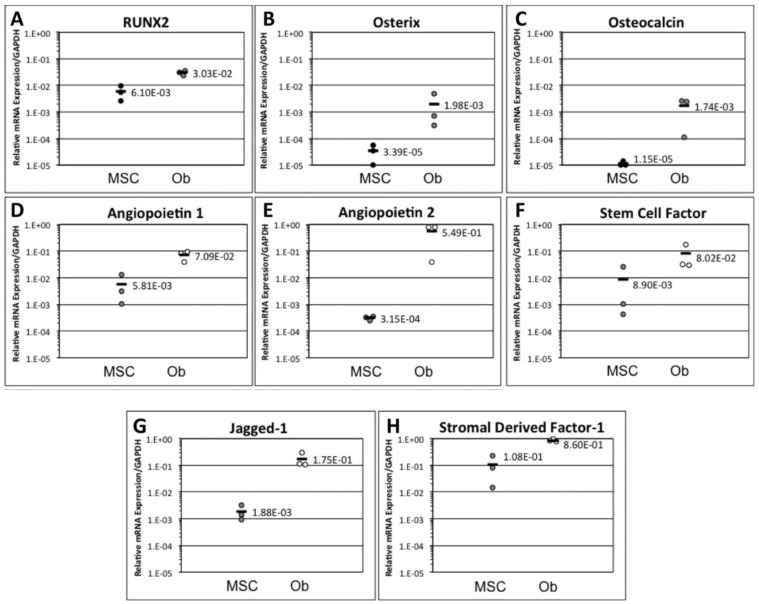
Comparison of mesenchymal stromal cell (MSC) and osteoblast monolayers for bone-related and haematopoietic stem cell (HSC) niche markers. Differentiation into osteoblasts was shown to increase the expression of (**A**) RUNX2, (**B**) osterix and (**C**) osteocalcin when compared to undifferentiated MSCs. MSCs or MSC-derived osteoblasts were examined for expression of HSC niche genes including (**D**) angiopoietin-1, (**E**) angiopoietin-2, (**F**) stem cell factor, (**G**) jagged-1 and (**H**) stromal cell-derived factor-1 (CXCL12). Data is shown as mean ± SD with each point representing independent MSC and MSC-derived osteoblast cultures from distinct mice.

The ability of MSCs to differentiate into osteoblasts was confirmed by qRT-PCR for lineage-specific markers. It was shown that MSC-derived osteoblasts expressed higher levels of markers for early osteogenic development, namely RUNX2 and osterix, when compared to undifferentiated MSCs (Figure 1A,B). Furthermore, MSC-derived osteoblasts expressed higher levels of osteocalcin (also known as bone gamma-carboxyglutamate protein), a marker of mature osteoblasts (Figure 1C) [12]. 

MSCs and osteoblasts were also assessed by qRT-PCR for their expression of HSC niche markers as an indication of their potential to support HSC growth and proliferation *in vitro*. A panel of niche-specific markers was selected which included angiopoietin-1, angiopoietin-2, stem cell factor, jagged-1 and SDF-1. All assayed genes were detected in both MSC and osteoblast populations; however, significantly higher expression of all genes was observed in osteoblasts (Figure 1D–H). This suggested that both populations had the propensity to support HSC growth *in vitro* but that osteoblasts might be a superior candidate for this. 

### 3.2. Osteoblasts Preferentially Induce Haematopoietic Cell Expansion Relative to Undifferentiated MSCs

**Figure 2:**
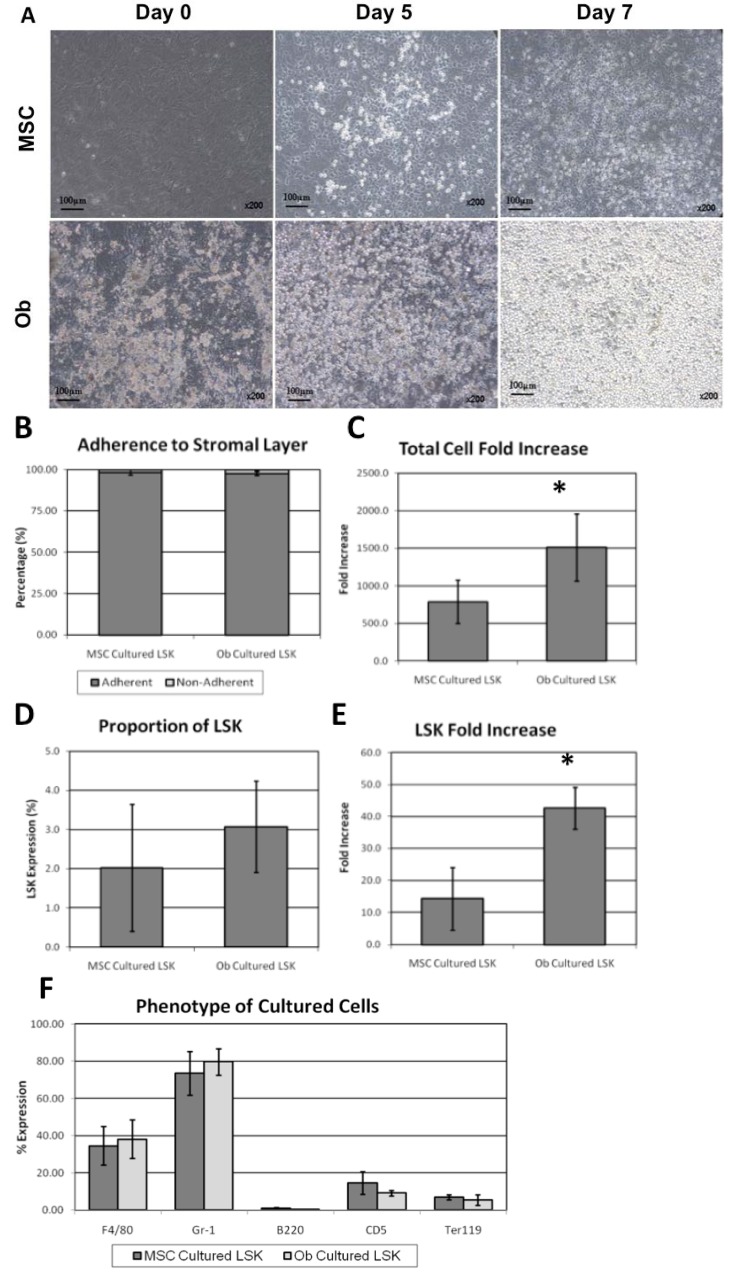
Haematopoietic cell expansion on stromal cell monolayers. (**A**) Co-cultures of HSCs with undifferentiated MSCs or osteoblasts after 0, 5 and 7 days by light microscopy; (**B**) Haematopoietic cells adhered to the stromal layer after the culture was washed (*n* = 8); (**C**) Total cell fold expansion after 7 days from the original 1000 LSKs was higher on osteoblast than on undifferentiated MSC monolayers (*p* *=* 0.0013; *n* = 8); (**D**) Flow cytometry of cultures revealed no differences in LSK proportion on either monolayer (*n* = 8); (**E**) Expansion of LSK HSCs, as determined by the product of total expansion and LSK proportion per culture, was significantly higher on osteoblasts compared to undifferentiated MSCs (*p* *=* 0.003; *n* = 8); (**F**) Myeloid, B cell, T cell and erythroid lineage commitment was similar when cultured on either stromal monolayer (*n* = 3). Data is shown as mean ± SD with each point representing independent co-cultures with from individually isolated LSK, MSC and MSC-derived osteoblast cultures from distinct mice.

For co-culture of LSKs on MSCs or osteoblasts monolayers, only serum was used: No haematopoietic growth factors or any other proteins were added. Co-cultures were optimised for haematopoietic cell expansion with X-vivo-10 medium supplemented with 20% FCS shown to be optimal for proliferation. 1000 LSKs were seeded on confluent MSC or osteoblasts monolayers. Haematopoietic cell expansion occurred on both undifferentiated MSCs and on osteoblasts (Figure 2A,B). After 5 days, cobblestone-like areas of adherent haematopoietic cells were observed (Figure 2A). After 7 days, over 95% of haematopoietic cells were found to be adherent to the stromal layer (Figure 2B). A mean 788-fold increase (±291-fold) in total haematopoietic cells on MSC monolayers was observed with an approximate two-fold higher total expansion (1513 ± 449-fold) when LSKs were co-cultured with osteoblasts compared to undifferentiated MSCs (*p* *=* 0.0013) (Figure 2C). This equated to a mean doubling time for haematopoietic cells on MSCs and osteoblasts of 17.5 h and 15.9 h respectively. The expansion appeared to be MSC- or osteoblast-specific, since LSKs co-cultured in the presence of adipocyte-differentiated MSCs showed no expansion of LSKs (data not shown), suggesting that marrow adipocytes are negative regulators of HSC expansion and/or survival, and confirming the findings of two previous studies [42,43]. Similar proportions of LSK cells were found to be present whether undifferentiated MSCs or osteoblasts were used (Figure 2D). However, as a result of the higher total cell expansion on osteoblasts, an increased yield of LSK was obtained in osteoblast/LSK co-cultures (*p* *=* 0.003) (Figure 2E). When characterising these expanded cells it was found that a majority of the expanded haematopoietic cells were lineage-committed (Figure 2F). The degree and type of haematopoietic lineage commitment was similar regardless of whether the supporting monolayer was of undifferentiated MSCs or osteoblasts, with a majority of the lineage-committed cells being of the myeloid lineage (Figure 2F).

### 3.3. MSC-Cultured HSC Engraft in Primary and Secondary Transplants Recipients while Osteoblast-Cultured HSCs are Associated with Poorer Haematopoietic Engraftment

**Figure 3:**
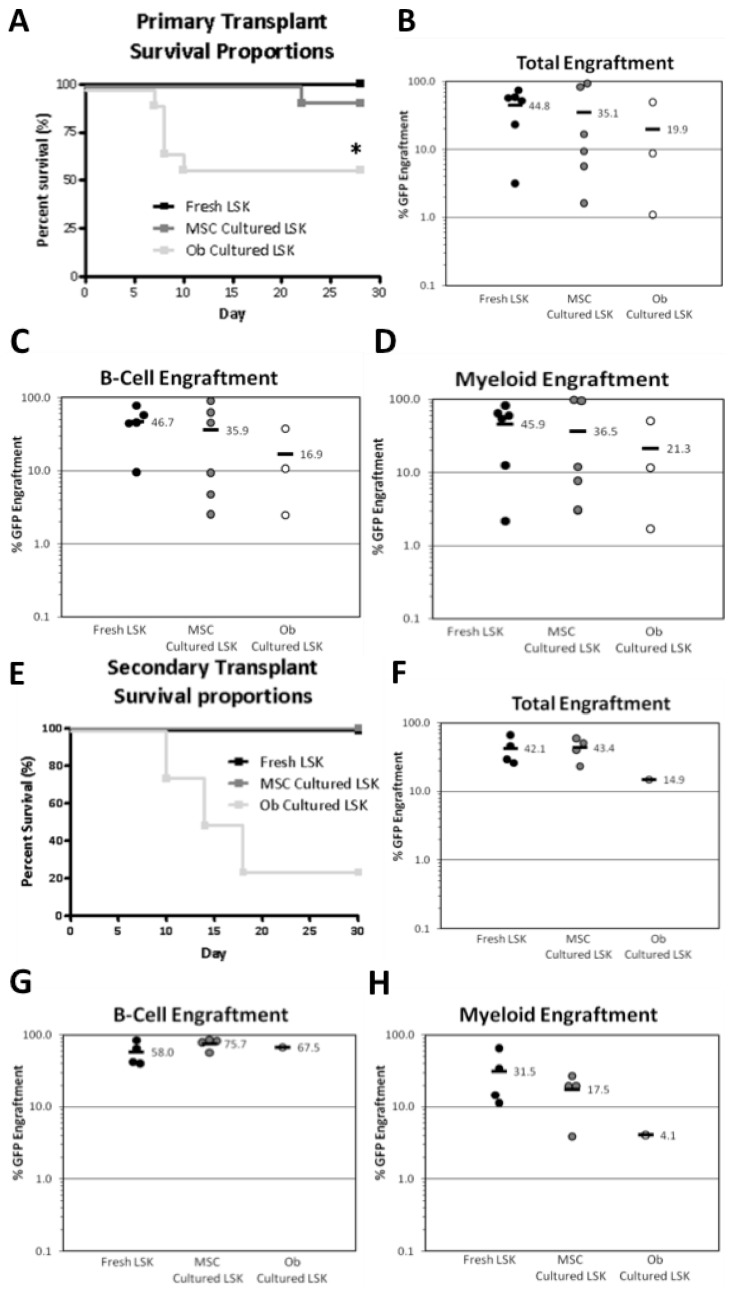
Primary and secondary transplantation of expanded LSKs into lethally myeloablated mice. Competitive repopulation transplant assays were performed in which the donor GFP^+^ co-cultured cells were co-transplanted with GFP^−^ competitor whole BM and compared to freshly isolated LSK transplants. (**A**) Primary transplantation of co-cultured cells at 4 weeks showed decreased survival in the osteoblast co-culture group (*p* = 0.0062; *n* = 12 pooled from 3 independent experiments). At 12 weeks post-transplant the proportion of GFP^+^ (**B**) total haematopoietic cells, (**C**) B cells and (**D**) myeloid cells was comparable in each group (*n* = 6 per group). (**E**) Secondary transplantation of pooled whole BM from primary recipients also showed markedly poorer survival at 4 weeks post-transplant in recipients of marrow in which the primary recipients had received LSKs co-cultured with osteoblasts compared to those receiving fresh LSKs or marrow from primary recipients that had received marrow co-cultured with undifferentiated MSCs. At 12 weeks post-transplant the proportion of GFP^+^ (**F**) total haematopoietic cells, (**G**) B cells and (**H**) myeloid cells was similar between recipients of fresh LSKs and secondary recipients of marrow in which the primary recipients had received marrow co-cultured with undifferentiated MSCs. Engraftment of marrow in which the primary recipients had received LSKs co-cultured with osteoblasts was not possible to assess since only 1 such mouse survived to this time point (*n* = 4 per group). (Mean ± SD).

CD marker profile/phenotype is not always indicative of cell function and thus flow cytometry data cannot be used to identify functional HSC numbers (*i.e.*, those with the ability to repopulate the BM). As such, an *in vivo* competitive repopulation model was used to compare the functional capacity of 200 LSK expanded on either undifferentiated MSCs or osteoblast monolayers to that of 200 freshly isolated LSKs. At 12 weeks after transplantation we found that LSKs co-cultured for 7 days on MSCs or osteoblasts contained functional HSCs after primary transplantation into myeloablated recipient mice (Figure 3B–D). There was no significant difference between the co-cultured cells and the freshly isolated LSK cells in these experiments (Figure 3B–D), indicating that functional HSCs had been maintained in co-cultures with MSC and osteoblasts despite the absence of exogenous cytokines. As an additional test for the functional capacity of putative HSCs, BM cells were taken from animals that had received a primary transplant of co-cultured LSKs and were transplanted into myeloablated syngeneic secondary hosts. At 12 weeks after total body irradiation and cell infusion mean multi-lineage haematopoietic reconstitution was similar to that following primary transplantation (Figure 3F–H). Multi-lineage reconstitution was again evident for total haematopoietic cells, B cells and myeloid cells (Figure 3F–H) at a similar level to the fresh LSK recipient controls. This reinforced the results from the primary transplants indicating that HSC were maintained during culture with MSC and osteoblast monolayers. However, HSC were not expanded, as engraftment was not increased. The fact that reconstitution in myeloid and B lineages was equivalent proves that these cultured LSK cells were capable of long-term multi-lineage reconstitution. If a bias in either myeloid or B lineage had been observed, the conclusion would be that HSC clonal subtypes with different differentiation potentials (such as α-HSC, β-HSC, γ-HSC and δ-HSC as defined by Benz *et al.*, 2012 [44]) were differentially selected on MSC or osteoblasts. This was not the case in the transplants shown. Therefore, co-cultures on MSC or osteoblasts are likely to maintain all HSC regardless of their clonal subtype. 

Surprisingly, mice receiving LSKs co-cultured on osteoblasts, while showing haematopoietic engraftment at similar levels to that of freshly isolated LSKs, displayed significantly poorer survival than those receiving LSKs co-cultured on MSC (*p* *=* 0.0062) (Figure 3A) due to haematopoietic failure between days 6–10 following transplantation. This was despite all mice receiving 4 × 10^5^ healthy competing BM cells, which theoretically contained 4–6 long-term repopulating HSCs (enough to ensure haematopoietic reconstitution and survival). Thus, we concluded that transplantation of LSK-osteoblast co-cultures compromised survival of recipients of the primary transplants and that this in turn compromised survival of recipients of the secondary transplants (Figure 3A,E respectively). This suggested that the self-renewal capacity of normal (GFP^−^) HSCs in the survivors of the primary transplants was compromised. 

We hypothesised that co-culture of LSKs on osteoblasts compromised HSC homing and engraftment. One possibility to explain this was that residual osteoblasts, harvested from the co-cultures, were trapped in the lungs and prevented HSCs accompanying them from reaching sites of haematopoiesis including the bone marrow and spleen. An alternative possibility was that the normal homing mechanisms of HSC was in some way impaired by their co-culture and administration with osteoblasts. 

### 3.4. MSC and Osteoblasts are Trapped in the Lungs Shortly after Transplantation

**Figure 4:**
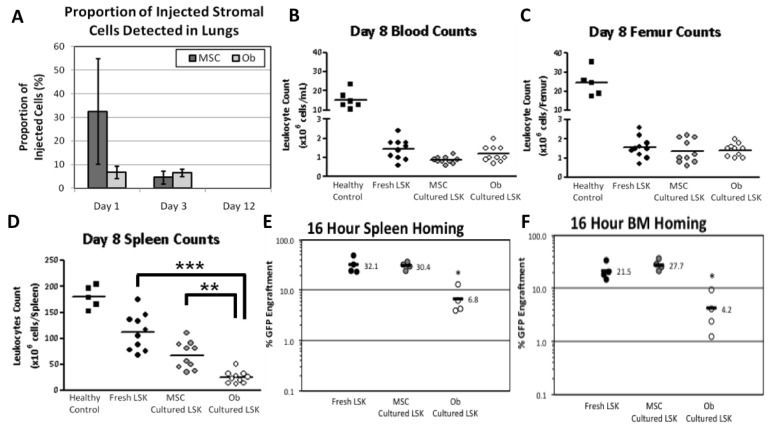
Early post-transplant migratory studies of MSC, osteoblasts and cultured LSKs. (**A**) GFP^+^ MSCs and osteoblasts were detected in the lungs by qPCR for GFP on days 1 and 3 but not on day 12 after primary transplantation (*n* = 4 per time-point). Co-cultured donor GFP^+^ leucocytes were detected in the (**B**) blood, (**C**) BM and (**D**) spleen at eight days post-transplant (*n* = 4 per group). Leucocyte counts in the spleen 8 days after primary transplantation showed a significant decrease in splenic leucocytes in the osteoblast co-culture group (*p* = 0.0084) compared to recipients of fresh LSK or recipients of marrow co-cultured with undifferentiated MSCs (*n* = 10 per experimental group pooled from 3 independent experiments; healthy controls *n* = 5–6). At 16 h postransplant, significantly less donor GFP^+^ haematopoietic cell homing was detected in recipients of osteoblast co-cultured LSKs in the (**E**) spleen and the (**F**) BM compared to recipients of fresh LSKs and MSC co-cultured LSKs (* *p* < 0.05; data presented from 1 transplant with 4 recipients per group). Data represent mean ± SD.

Using MSCs from mice transgenic for luciferase, we have previously shown that intravenously injected MSCs are detectable in the lungs within minutes of injection [45]. Thus, we hypothesised that large, matrix-secreting osteoblasts bind HSCs tightly prior to injection and that theses complexes becomes lodged in the small capillaries of the lungs, thus preventing optimal homing of both expanded and competitor-repopulating HSCs to sites of haematopoiesis. To explore this hypothesis, GFP^+^ osteoblasts or GFP^+^ MSCs were injected intravenously into lethally irradiated C57BL/6 recipients with unlabelled co-cultured haematopoietic cells and the lungs harvested at 1 day, 3 days or 12 days post-injection. GFP^+^ MSCs and GFP^+^ osteoblasts were detected by qRT-PCR for genomic GFP in harvested lung tissue at 1 and 3 days after injection, but were not detectable at 12 days after injection (Figure 4A). These findings are very similar to those using luciferase transgenic MSCs [45]. 

### 3.5. Transplantation of LSK/Osteoblasts Co-Cultures Compromises Short-Term Splenic Reconstitution

We next investigated whether MSC and osteoblast entrapment in the lungs was preventing HSC migration to the marrow and spleen. This was performed in a similar manner to the primary transplants (Section 3.3) by transplanting freshly isolated GFP^+^ LSKs or GFP^+^ LSKs co-cultured with unlabelled MSCs or osteoblasts together with whole BM competing cells. Assessment of leucocyte counts in the blood, BM and spleen was performed at the typical time of death in the osteoblast co-cultured transplant group (day 8–14 post-transplant). This revealed no observable differences between these groups in cellular reconstitution of blood or BM at day 8 post-transplant (Figure 4B,C). However, at day 8 post-transplant significantly fewer leucocytes were found in the spleens of recipients that had received HSCs co-cultured with osteoblasts compared to recipients of fresh LSKs (*p* *<* 0.001) and LSK/MSC co-culture recipients (*p* *<* 0.01) (Figure 4D). Since this was a competitive transplant experiment, it appeared that osteoblasts from the co-culture prevented both co-cultured HSCs and competitive unmanipulated HSCs from reaching the spleen and proliferating there. Thus, we concluded that the cause of death in the osteoblast/LSK co-cultured recipients was due to an impairment of short-term reconstitution, particularly in the spleen, rendering recipients susceptible to pancytopenia and subsequent infection. We next explored the question of homing to sites of haematopoiesis (marrow and spleen) of these different populations.

### 3.6. Impairment of Homing Ability to the Spleen

The above experiments did not show whether the impairment of short-term splenic reconstitution was due to compromised splenic proliferation of haematopoiesis or compromised homing to the spleen. However, since this was a competitive transplant model (*i.e.*, recipients were co-transplanted with 4 × 10^5^ unmanipulated BM cells—A dose that normally allows short- and long-term repopulation), we hypothesised that this was due to compromised homing of both transplanted populations. To explore this, we assessed the homing of fresh GFP^+^ HSCs, LSKs co-cultured with MSCs and LSKs co-cultured with osteoblasts to the spleen at 16 h post-transplant, a time point at which detectable proliferation was unlikely. This revealed that the transplantation of LSK/osteoblasts co-cultured cells led to significantly reduced homing to the spleen (*p* = 0.0245) when compared to fresh LSK and LSK-MSC co-culture recipients (Figure 4E). Similar findings were made for BM homing (Figure 4F, *p* = 0.0154). In conjunction with the low splenic leucocyte counts, this implied that the transplanted competitive BM cells with repopulation ability cultured on osteoblasts did not reach the spleen early post-transplant. This led to complete failure of homing and engraftment of HSC in 50% of the animals and death within 10 days of transplant (Figure 3A). The other 50% of recipients survived for the duration of the study and showed a similar BM reconstitution to that of fresh LSK and MSC-cultured LSK (Figure 3B–D). Hence, we propose that the co-transplantation of osteoblasts significantly inhibits and/or prevents migration of cells with repopulation ability to both the spleen and BM. In order to attempt to confirm this, we then removed osteoblasts from the co-culture prior to injection of the co-cultured LSK.

### 3.7. Removal of Osteoblasts from Co-Cultures Prior to Injection is Required for Short-Term Reconstitution

**Figure 5:**
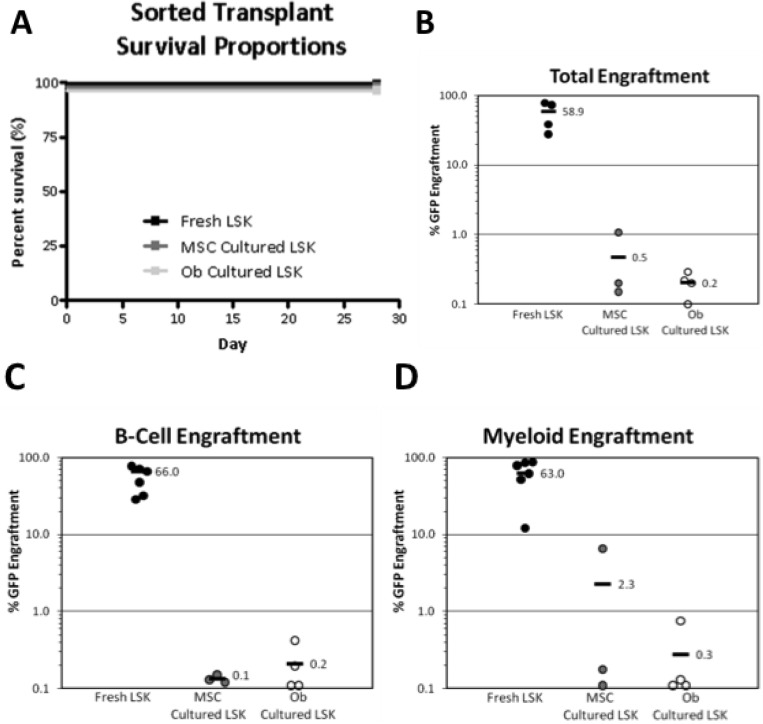
Primary transplantation of co-culture LSKs after removal of stromal cells. Co-cultures were initiated with GFP^+^ LSKs and unlabelled MSCs or osteoblasts. After harvest of cultures, flow cytometry sorting was performed and only cells expressing GFP, containing cultured haematopoietic cells but devoid of undifferentiated MSCs or osteoblasts, were selected. These were then transplanted into myeloablated primary recipients. (**A**) Four week survival was 100% in each group. However, the proportion of GFP^+^ LSKs contributing to total haematopoietic cells in the (**B**) blood, (**C**) B cells and (**D**) myeloid cells was significantly less compared to that demonstrated by fresh LSKs. (*n* = 4 per group; data represent mean ± SD).

Seven day co-cultures of GFP^+^ LSKs with unlabelled MSCs or with osteoblasts were initiated as before. The total resulting cells were harvested and the GFP^+^ haematopoietic cells were isolated by FACS sorting. These cells were then transplanted with whole BM competitors as before. Transplants depleted of osteoblasts now showed 100% survival at 30 days postransplant—The same result as with fresh LSKs and LSKs cultured with undifferentiated MSCs (Figure 5A). These findings showed that removal of osteoblasts from cell suspensions prior to infusion allowed short-term reconstitution. However, removal of MSCs or osteoblasts before transplantation paradoxically compromised the engraftment of the co-culture-expanded donor GFP^+^ haematopoietic cells (Figure 5B–D). Only a single mouse showed donor GFP^+^ myeloid engraftment in the MSC-cultured LSK transplanted group (Figure 5D). This suggested that HSC were lost during the sorting process. Since the cells were mono-dispersed prior to sorting, this loss was likely due to shear damage in the sorting process. Alternatively, the removal of osteoblasts may have allowed the “released” LSKs to differentiate more and thus lose their self-renewal potential.

## 4. Conclusions

In this study we show that purified murine LSKs, a population enriched for HSCs, can be co-cultured with both undifferentiated MSCs or osteoblasts differentiated from MSCs, without the addition of exogenous growth factors and that this can maintain haematopoietic cells which can subsequently contribute to long-term *in vivo* haematopoiesis in myeloablated recipient mice after both primary and secondary transplantation. However, while both cell types were shown to maintain the number of engrafting HSCs, neither could expand them as demonstrated by *in vivo* competition repopulating assays. Cells of the osteoblast lineage have previously being shown to be key regulators of the HSC niche [18,20] and thus would appear a good candidate to support *ex vivo* HSC growth for clinical transplantation purposes, particularly in settings in which timely haematopoietic engraftment is problematic such as after cord blood transplantation in adults [3]. Recently, endothelial cells have been shown to be essential to HSC maintenance and proliferation within the BM niche [24,25,26]. However, the co-cultures in this paper did not include endothelial cells and this could provide an explanation as to why HSCs could only be maintained rather than expanded in this system.

When comparing co-culture of an undifferentiated MSC monolayer to that of a monolayer of osteoblasts derived from undifferentiated MSCs, osteoblast co-cultures led to a significantly greater increase in cells with the LSK phenotype (over 40-fold from their original starting number). Conversely, however, when LSKs were seeded into cultures with adipocytes derived from MSCs, no growth was observed. This is supported by recent data suggesting that adipocytes have a negative influence on HSC growth [42,43], although a third report demonstrated the opposite effect [46]. Haematopoietic cells adhered to both MSCs and osteoblasts in co-culture, consistent with cobblestone area-forming cells (CAFC) and long-term culture-initiating cell (LTC-IC) assays that have been developed as surrogate *in vitro* assays in attempts to determine true HSC qualities. Moreover, it has also been shown that the more primitive HSC/HPC populations (as identified through LTC-IC assays) have a higher affinity for stromal monolayers than their committed progeny [29,30,31].

In this study we have described for the first time the paradoxical effect of higher *in vitro* numbers of haematopoietic cells co-cultured with osteoblasts but poorer *in vivo* survival in competitive serial haematopoietic cell transplantation assays with such LSKs compared to those co-cultured on undifferentiated MSCs. This appeared to be due to poorer homing and, presumably, subsequent proliferation, of the osteoblast co-cultured LSKs on reaching sites of haematopoiesis after intravenous injection into the myeloablated hosts.

No significant difference in phenotype was observed with either MSC or osteoblast co-cultured HSCs after a seven-day expansion period. Consistent with previous studies, the majority of the resulting cells appeared committed to the myeloid lineage (Gr-1^+^ or F4/80^+^). Very low proportions of lymphoid and erythroid cells were found. These data support the potential of such a system for granulocyte expansion as prevention or treatment of neutropenia.

We demonstrated the functional capacity of LSKs co-cultured on undifferentiated MSCs or osteoblasts using in an *in vivo* competitive repopulation transplant model. We showed that overall engraftment capacity in primary recipients of LSK co-cultured on undifferentiated MSCs was equivalent to that of freshly isolated LSKs both for multi-lineage reconstitution and for LSK engraftment. Although there was an increase in LSK cell number by phenotype (*i.e.*, by flow cytometric analysis) within LSK-MSC co-cultures, the numbers of HSC with true engraftment and repopulation potential were maintained rather than expanded. This effect was further verified in serial transplants where donor cells from primary recipients of LSKs co-cultured on undifferentiated MSCs were able to repopulate secondary hosts at a similar efficiency to that of freshly isolated LSKs. In contrast, however, recipients of LSK co-cultured on osteoblasts displayed significantly lower survival rates after primary *in vivo* transplantation. This result was both surprising and perplexing as the model used to test the functionality of the cultured cells was a competitive transplant model, meaning that hosts received both manipulated HSCs from the expansion cultures and unmanipulated, freshly isolated HSCs from whole BM. Thus, even if functional HSCs were not maintained in LSK/osteoblast co-cultures, hosts receiving these transplants should still be have been repopulated by the whole BM competing cells. Furthermore, when engraftment in the primary recipients did occur, self-renewal capacity remained compromised as demonstrated by the significantly lower survival observed after secondary transplantation. To explain this, we hypothesised that osteoblasts transplanted concurrently with the donor and competitor haematopoietic populations, adversely influenced the fate of their co-transplanted HSCs. Therefore, we investigated whether a factor present in the LSK/osteoblast co-cultures was either directly causing death, or indirectly preventing both co-cultured HSCs and competing whole BM from homing to, and engrafting in, the marrow and spleen. 

We further hypothesised that osteoblasts, after intravenous infusion, got trapped in the endothelial capillaries of the lungs, causing pulmonary embolism and subsequent pulmonary ossification post-transplant. We have previously shown that undifferentiated MSCs are trapped in the lungs after intravenous infusion [45]. This was also the case in this study (using stromal cells transgenic for GFP rather than MSCs from luciferase-transgenic mice [45]), in which it was found that both undifferentiated MSCs and osteoblasts were trapped in the lungs for up to 3 days post-transplant. However, these cells not detected in the lungs at 12 days post-transplant, a typical time of death in these recipients. It thus seemed unlikely that osteoblasts were causing significant pulmonary pathology after their intravenous injection. We considered whether transient osteoblast entrapment in the lungs was compromising long-term engraftment ability of the co-injected LSKs. However, this appeared unlikely as more undifferentiated MSCs than osteoblasts were found in the lungs without engraftment being compromised. 

As indicated above, we noted that the time of death was consistent with that caused by haematopoietic failure in lethally myeloablated recipients (~days 8–14 post-irradiation). Leucocyte counts in the blood, femoral BM and spleen were analysed at day 8 post-transplant. Very few leucocytes were found in the blood or BM, as is typical after myeloablation and there was no significant difference between the different co-cultured groups. However, recipients of osteoblast co-cultured LSKs had significantly fewer leucocytes in the spleen and significantly lower donor cell engraftment in the BM and spleen. Thus, the cause of mortality in these animals was likely due to delayed short-term reconstitution in the spleen with subsequent anaemia and/or infection due to pancytopenia. The reason for this haematopoietic failure was next explored: Donor cell homing to the spleen and BM was investigated at 16 h post-transplant. This showed that significantly lower numbers of donor cells from LSK/osteoblast co-cultures homed to the spleen and BM and that virtually all engraftment was from the competitive whole BM population in these organs. In secondary transplants, these recipients still demonstrated 50% mortality, indicating that co-transplantation of osteoblasts significantly inhibited the proliferation of all cells with repopulation ability, from both the donor and competitor populations, in the spleen and BM.

Finally, we showed that transplantation of culture suspensions devoid of stromal cells (physically removed prior to transplant) prevented the mortality observed in recipients of osteoblast co-cultured LSKs. However, while the competitor HSCs successfully engrafted in this case, the repopulation ability of the co-cultured LSKs was compromised and almost all engraftment was from the competing whole BM populations. This indicated that removal of stromal cells from culture suspensions also removed the HSCs with long-term repopulating characteristics. Thus, we have identified a confounding feature that may exist in culture systems that contain osteoblasts and this emphasises the importance of pre-clinical HSC transplant models to identify such limitations.

Overall, this co-culture assay has enabled comparison of undifferentiated MSCs and osteoblasts to support HSCs in culture. The system will be useful to further identify constituents that may be used to enhance *ex vivo* expansion of HSC, and to translate it to the human setting using CD34^+^ cord blood HSCs co-cultured with human MSCs or their differentiated progeny.
